# Intersection of Wildfire and Legacy Mining Poses Risks to Water Quality

**DOI:** 10.1021/acs.est.4c09489

**Published:** 2024-12-19

**Authors:** Sheila F. Murphy, Johanna M. Blake, Brian A. Ebel, Deborah A. Martin

**Affiliations:** † U.S. Geological Survey, Water Resources Mission Area, 3215 Marine Street, Boulder, Colorado 80303, United States; ‡ U.S. Geological Survey, New Mexico Water Science Center, 6700 Edith Blvd. NE, Albuquerque, New Mexico 87113, United States; § U.S. Geological Survey, Water Resources Mission Area, Burlington, Vermont 05482, United States

**Keywords:** wildland fire, metals, disturbances, water supplies, western United States, compound events, geochemical hazards

## Abstract

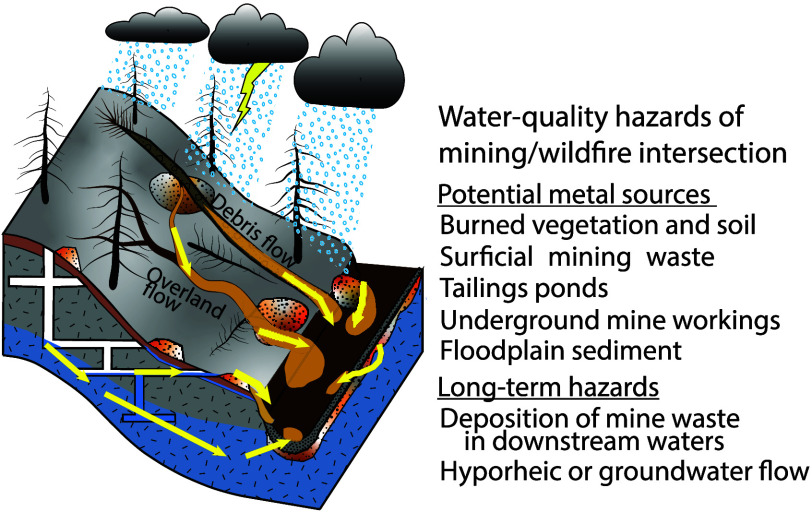

Mining and wildfires are both landscape disturbances that pose elevated and substantial hazards to water supplies and ecosystems due to increased erosion and transport of sediment, metals, and debris to downstream waters. The risk to water supplies may be amplified when these disturbances occur in the same watershed. This work describes mechanisms by which the intersection of mining and wildfire may lead to elevated metal concentrations in downstream waters: (1) conveyance of metal-rich ash and soil to surface waters, (2) increased dissolution and transport of dissolved metals due to direct contact of precipitation with mine waste, (3) increased erosion and transport of metal-rich sediment from mining waste, (4) remobilization of previously deposited metal-contaminated floodplain sediment by higher postfire flood flows, and (5) increased metal transport from underground mine workings. Predicted increases in wildfire size, frequency, and burn severity, together with the ongoing need for metal resources, indicate that improved mapping, monitoring, modeling, and mitigation techniques are needed to manage the geochemical hazard of the intersection of wildfire and mining and implications for water availability.

## Abstract 2

Wildfires in legacy mining areas pose hazards to downstream water quality due to increased erosion and transport of sediment and metals to surface water.

## Introduction

Water scarcity is an increasing concern globally.^1,2^ Impairment of water for its intended usage has been identified as a type of water scarcity,^3−5^ and, thus, water-quality deterioration can exacerbate water-supply shortages^6^ and harm ecosystem health.^7^ Hydroclimatic extremes and climate shifts can be primary drivers of surface water quality decline.^7,8^ Climate drivers in combination with landscape disturbances and other hazards, termed “compound events,” can be particularly deleterious to water quality.^9,10^ Despite synergistic process interactions that can cause severe effects on water quality in response to compound events, traditional risk assessments consider single drivers in isolation, potentially underestimating hazards to water quality.^10^ As compound events become increasingly common^11,12^ and the subsequent water-quality effects are recognized,^13−15^ guidance for land, water, and ecosystem management will need to include the effects of overlapping climate extremes and landscape disturbances.

Mining and wildfires are two major disturbances that can have substantial impacts on downstream water quality. Water discharged from mines can have extreme pH and (or) be rich in metals (and metalloids, grouped here with metals),^16^ and metal-rich mine waste can be transported 10s to 100s km downstream and stored for extended periods (1,000–100,000 years) in floodplains and lake sediments.^17−22^ Mining-affected floodplains are now the primary source of metals to rivers in the United States (U.S.) and western Europe.^22^ These metals are being remobilized by floods,^19,22,23^ and due to predicted increases in rainfall intensity, flooding-driven redistribution of mining-affected floodplain sediment will likely worsen in the future.^18,24,25^ Wildfires can lead to enhanced erosion and sediment transport and subsequent increases in sediment, nutrients, and metals in downstream waters,^26,27^ resulting in stream habitat degradation and inflated water treatment costs.^5,28,29^ Downstream effects may extend for 100s of km^30−32^ and last more than a decade.^29,33−35^ Modeled projections suggest that a third of western U.S. watersheds will have >100% more sedimentation by 2050 because of wildfire.^36^

**Figure 1:**
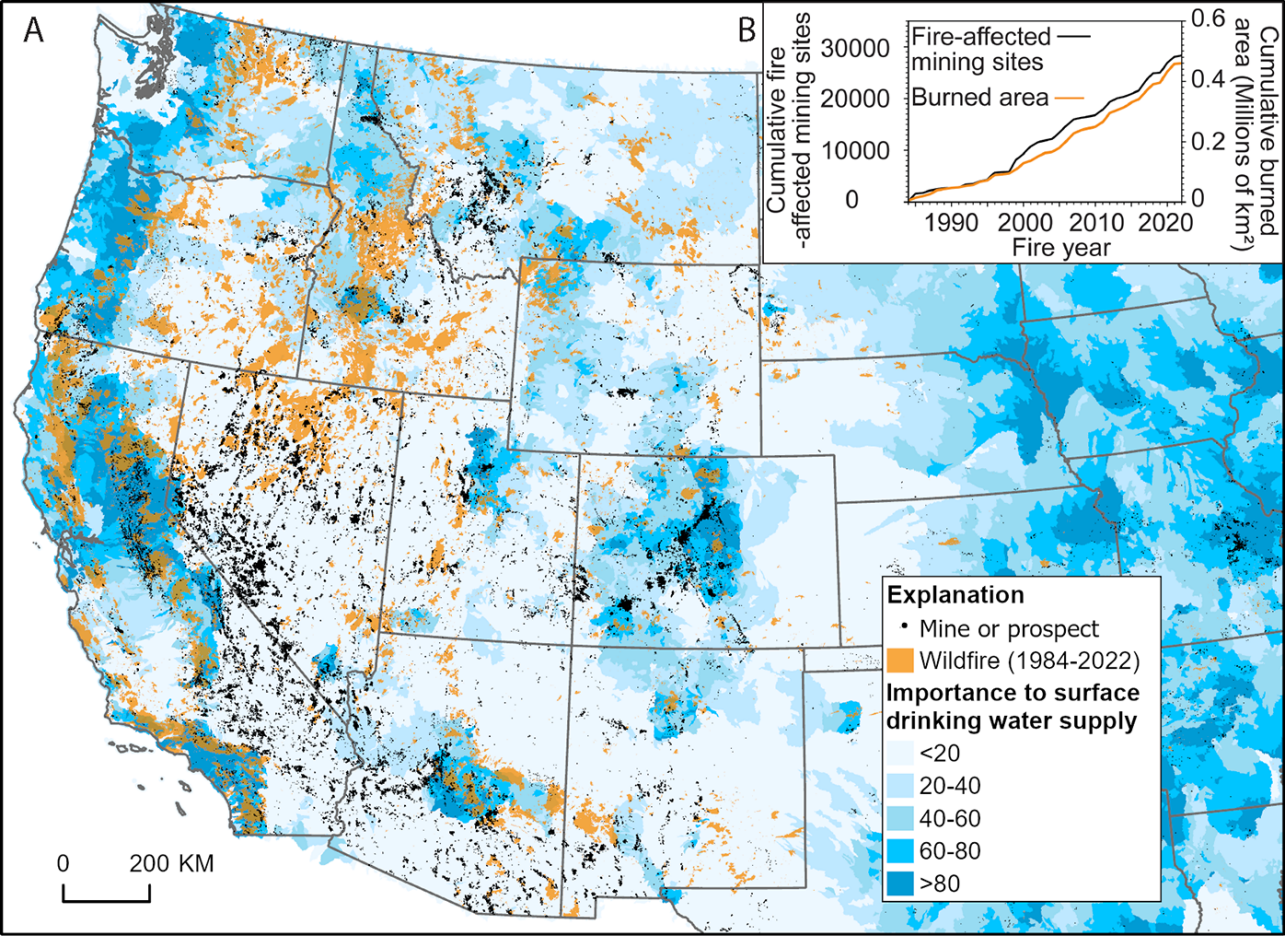
(A) Map of the western U.S. showing mines and prospects (excluding gravel, sand, and borrow pits and quarries),^131^ wildfires (1984–2022),^132^ and an index of relative importance to surface drinking water (based on average annual water yield multiplied by a drinking water protection model that includes population served and intake locations)^133^ and (B) graph showing cumulative area burned^134^ and cumulative number of wildfire-affected mining sites in the western U.S. (intersection of wildfire perimeters^134^ and point locations of mine sites;^131^ western U.S. here refers to the states of Arizona, California, Colorado, Idaho, Montana, Nevada, New Mexico, Oregon, Utah, Washington, Wyoming).

Wildfires are now burning in mining-affected watersheds in many areas of the world, including North America,^37−41^ South America,^42^ Australia,^43−46^ Europe,^47^ Asia,^48^ and Africa.^49,50^ In the western U.S., the intersection of these hazards often coincides with important surface water supply watersheds^39,40,46^ (Figure 1), with substantial water-quality and land management implications. For example, wildfire in a legacy mining area in Colorado led to elevated stream concentrations of arsenic for at least five years after the fire^39^ and required removal of a ∼70-year-old arsenic- and lead-rich tailings deposit to protect downstream water supplies.^51^ A postfire debris flow in Montana mobilized mine waste into a stream, leading to a multimillion-dollar cleanup effort.^38,52^ Observed and anticipated increases in extreme wildfire behavior and severity,^53^ in concert with amplification of storm intensity in many parts of the world,^54,55^ suggest that the intersection of wildfire and mining and subsequent risk to water supplies may worsen. Previous work described potential pathways of metals to surface water in areas affected by wildfire and legacy mining.^39^ The objective of this work is to connect current concepts about remobilization of legacy mine waste^18,22,56−58^ with recent advances in understanding how wildfire affects landscapes and thus the erosion, mobilization, and transport of sediment.^59−62^ We also illustrate the hazards to water supplies posed by the wildfire-accelerated mobilization of mining-derived metals in the western U.S. and identify challenges and opportunities for targeted research. Finally, we explore the concept that overlapping landscape disturbances, such as mining and wildfire, may create conditions in receiving waters that are primed to respond disproportionately to extreme climate events, with substantial implications for water quality.

## Effects of Wildfire and Mining on Landscapes and Metal Mobilization

Both wildfire and mining can substantially alter geomorphology, vegetation, hydrology, and geochemistry, leaving landscapes at a greater risk of erosion and transport of sediment and metals (Table 1). Metal mining often results in the disposal of large amounts of coarse waste rock and fine-grained tailings on the landscape or into water bodies. While large-scale modern mining typically involves reclamation to reduce remobilization of waste, historical mining commonly entailed little to no remediation.^21^ Mine waste often has unnatural angles of repose, lacks soil structure, is enriched in metals, and is depleted of nutrients (Table 1). As a result, mine waste can remain devoid of vegetation for many years and is highly vulnerable to erosion by wind, water, or gravity (e.g., dry ravel and mass movement).^63−66^ Local surface and subsurface flow paths can be highly altered due to compaction, artificial stratification, and discontinuities in permeability.^25,67^

**Table 1:** Potential Vegetation, Geomorphological, Geochemical, and Hydrological Changes to Landscapes by Mining and Wildfire, and Mechanisms That May Lead to Elevated Metal Concentrations in Downstream Waters Caused by Their Intersection^a^

type of change	mining	wildfire
Vegetation		
	Removal of vegetation	Loss or reduction of vegetation
	Deposition of metals on surrounding vegetation and soil (dust and ore processing)	Conversion of vegetation and necromass to ash and charred debris
	Reduced canopy interception of precipitation	Reduced canopy interception of precipitation
	Minimal regrowth of vegetation; possibly revegetated with nonnative species	Gradual regrowth of vegetation; potential conversion to different vegetation type
Geomorphological		
	Decreased particle size, increased surface area	Decreased or increased particle size
	Reduced soil structure	Loss of aggregate stability and soil cohesion
	Increased susceptibility to erosion	Increased susceptibility to erosion
	Unnatural angle of repose	
Geochemical		
	Enriched in metals	Potentially enriched in metals
	Often extreme pH (very low or very high)	Ash can have high pH
	Low nutrient status	Often enriched in nutrients (depending on burn severity)
	Altered solubility and oxidation states of metals	Altered solubility and oxidation states of metals
		Change in composition and reactivity of organic carbon
Hydrological		
	Reduced infiltration	Reduced infiltration
	Shift to surface and near-surface flow during storms	Shift to surface and near-surface flow during storms
	Reduced evapotranspiration from vegetation removal	Reduced evapotranspiration from vegetation mortality

Effects of the intersection of mining and wildfire on metal mobilization and transport
Conveyance of metal-rich ash and soil to surface waters
Increased dissolution and transport of dissolved metals due to direct contact of precipitation with mine waste
Increased erosion and transport of metal-rich sediment from mining waste
Remobilization of previously deposited metal-contaminated floodplain sediment by higher postfire flood flows
Increased metal transport from underground mine workings

a Sources: refs (25, 33, 39, 59, 68−78).

Wildfires can partially or completely combust vegetation canopy, surface organic cover, and soil organic matter (Table 1), leading to the alteration of the chemical and physical properties of these materials.^68,69,73,79−81^ For example, wildfire ash can contain pyrogenic organic matter^34^ and (or) carbonates,^81^ and be enriched in metals such as manganese, lead, and zinc.^69^ Heating during wildfire can change the oxidation states of metals (such as arsenic and chromium),^82−84^ which will affect metal mobility, bioavailability, and toxicity. Depending on the temperature and duration of the fire, metals in vegetation, necromass, and soil may be released to the atmosphere or retained on the landscape,^46,68,69^ where they are vulnerable to redistribution by the same geomorphic processes that mobilize mining waste, i.e., water, wind, or gravity.^59,78,85,86^ In contrast to mine waste, erodible material left after wildfire, such as ash, soil, and partially burned organic matter, can be rich in nutrients such as nitrogen and phosphorus,^68^ which can have direct effects on water quality individually and through complexes with metals when transported to water bodies.^26^

Altered hydrologic flow paths brought about by both wildfire and mining (Table 1) lead to increased overland flow, higher flood peaks, shorter lag times between rainfall and flood peak, and higher sediment loads delivered to downstream waters.^74,87−89^ The highest risk of mobilization and transport of dissolved and particle-sorbed metals after either disturbance is during episodic, low-frequency, high-magnitude storm events,^26,63,90^ particularly in the years immediately after the disturbance.

## Potential Pathways of Metals from Mine Waste to Surface Water after Wildfire

Landscape conditions in the western U.S. are primed for enhanced metal mobilization by wildfires affecting previously mined lands (Figure 1). Wildfire is a risk for metal remobilization in mining areas if vegetation on or upstream of mine waste burns at a severity high enough to change hydrology or the character of erodible material.^13,39^ Metals may also be remobilized by wildfire if burned vegetation and necromass contained metals related to mining activities (such as atmospheric deposition from ore processing or bioaccumulation of metals from mine waste).^41^ Large mining sites often dominate public perception of mining, but they are slow to revegetate, particularly with forest, the land cover with the greatest postfire erosion hazard.^91^ In contrast, smaller, dispersed mine waste sites in areas that have been reforested are more vulnerable to wildfire and subsequent metal mobilization. Such small-scale prospects and dispersed mining sites are pervasive in the western U.S.^70^ (Figure 1).

**Figure 2:**
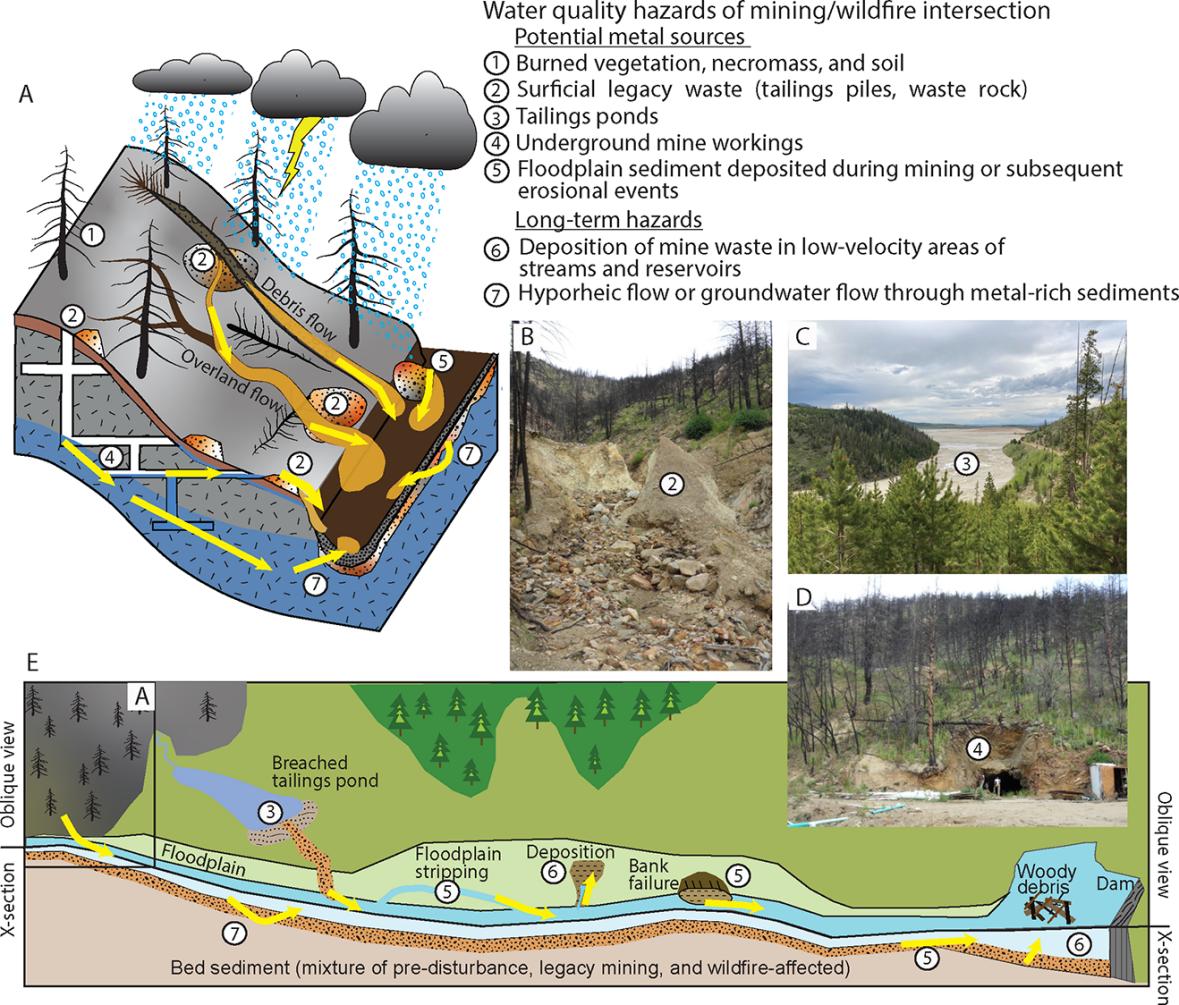
Conceptual diagram of water-quality hazards from the intersection of wildfire and mining. Number labels identify metal contamination sources and long-term hazards in diagrams and photographs. Yellow arrows denote pathways of dissolved and particulate metals. (A) Potential hillslope and headwater metal sources. (B) Mine waste within the 2010 Fourmile Canyon Fire burned area, Colorado, eroded by postwildfire floods. (C) Mine tailings impoundment in Grand County, Colorado. (D) Mine adit within the 2010 Fourmile Canyon Fire burned area, Colorado. (E) Conceptual model of downstream metal contamination sources and long-term hazards showing inset A in the basin headwaters. Photographs by Sheila Murphy.

The intersection of mining and wildfire may lead to elevated metal concentrations in surface waters via several mechanisms (Table 1, Figure 2):

- *Conveyance of metal-rich ash and soil to surface waters*. Burning of vegetation and necromass enriched in metals due to atmospheric deposition during historical ore roasting or smelting^41^ or to uptake from mineralized soils^92−94^ can result in metal-rich ash being readily available for mobilization and transport to downstream waters.^93,95^
- *Increased dissolution and transport of dissolved metals due to direct contact of precipitation with mine waste*. Precipitation falling on mine waste can lead to dissolution of metals from efflorescent salts.^56,96,97^ In areas where mine waste had been sheltered from direct precipitation, either by revegetation or interception by adjacent trees, the re-exposure of metal-rich mine waste due to wildfire-induced vegetation mortality will increase the contact of precipitation with efflorescent salts, and overland flow paths can transport the metals to streams.^39^
- *Increased erosion and transport of metal-rich sediment from mining waste*. Substantial direct erosion of mine waste has been observed at many sites during heavy rainfall,^18,19,23,39,63^ and wildfire-induced vegetation mortality will increase susceptibility to rain-driven erosion (Figure 2). Wildfire typically reduces infiltration, leading to decreases in the threshold rainfall rate required for overland flow.^90,98^ Even in areas where revegetation on mine waste is minimal, burning of upgradient forest could increase overland flow moving over or adjacent to the mine waste, accelerating erosion (Figure 2). Because legacy tailings piles are often located at the base of hillslopes and proximal to stream channels, they are particularly vulnerable to increased remobilization during high-flow events.^18,39^ Moderate-intensity postfire rainstorms have remobilized metal-rich legacy mine tailings in Colorado^39^ and Montana.^52^ However, increased erosion in burned areas upstream of mining waste could temporarily dilute metal concentrations by delivering alkaline ash, metal-poor sediment, and freshly exposed mineral surfaces, leading to increased pH and precipitation or sorption of metals.^43,47,99^ Failure of tailings pond dams is a known environmental problem;^100^ wildfire-accelerated delivery of water and sediment to such impoundments could increase the risk of breaching.
- *Remobilization of previously deposited metal-contaminated floodplain sediment due to higher postfire flood flows*. Remobilization of metal-contaminated floodplains is becoming a greater problem due to increased flooding related to climate change and is likely to worsen.^18,22^ Alteration of watershed characteristics by wildfire typically leads to elevated peak flows,^87,101^ which can erode riverbanks and floodplains.^102^ Postfire flooding will likely increase the downstream dispersion of metal-enriched sediment, extending the extent of mining impacts with potential implications for remediation.
- *Increased metal transport from underground mine workings*. Increased metal concentrations in waters discharged from underground mine workings have been observed during storm events.^103,104^ Wildfire-induced shifts to overland flow could mean additional water moving into and through mine openings during storm events. In addition, wildfire-induced vegetation loss can reduce transpiration, resulting in more intrastorm subsurface flow,^105^ which could increase the amount or fluctuation of water moving through underground mine workings.^39^ Altered water movement through mine workings can change pH, oxidation state, and mineral stability, leading to dissolution of sulfide minerals and precipitation-redissolution of efflorescent salts.^57,103,106^ However, the effects of water flowing through mine tunnels are complex and poorly documented, and, while metal concentrations in mine effluent can increase, studies have also documented decreases.^103,107^

## Long-Term Risk to Downstream Waters

Downstream deposition of metal-rich sediment, together with wildfire ash, soil, and unburned vegetation, may have long-term implications for receiving waters. Sediment-laden waters eventually settle on floodplains or in slower-moving waters (Figure 2). Rapid accumulation and burial of carbon-rich sediments left after postfire flooding can lead to strongly reducing conditions below the sediment-water interface and subsequent dissolution of metal-bearing iron and manganese oxides and hydroxides.^39,108−110^ In addition, there may be changes to pH, presence of organic and inorganic ligands, and microbial activity, which influence metal speciation and bioavailability.^108−111^ This redeposited sediment is vulnerable to later remobilization by high flows, seasonal cycles of exposure and submergence, bioturbation, and dredging.^57^ While the greatest risk of flooding and water-quality impairment is typically within the first few years, the risk of elevated hydrologic and erosional responses can persist for longer periods,^112,113^ especially in response to extreme storms.^39^ Hyporheic exchange through mixtures of mining-derived sediments and postwildfire flood-derived sediments rich in charred material may pose additional water-quality hazards (Figure 2). Elevated metal concentrations and loads may have negative effects on downstream water supplies and ecosystems.^5,27,29,72^ The wildfire-mining combination can also increase the dispersion of mining waste and increase the length of the river channel that must be evaluated in environmental risk assessments. As these metal-rich sediments become further distributed in the downstream landscape, vulnerability to compound events like subsequent extreme rainfall may be exacerbated above the already high risks from legacy mining alone.^18^ Thus, the long-term risk to water quality from the deposition of both wildfire and mining debris in streambeds, floodplains, and reservoirs could be considered a “chemical time bomb”^114^ or “delayed geochemical hazard”.^115^

## Opportunities

There are many opportunities for improving our understanding and management of legacy mine waste in wildfire-prone regions:

- *Improved mapping of mining waste locations, extent, and character in forested areas at risk of wildfire*, *and relation to water supply watersheds and intakes*(116)*on a global scale*. Estimates of the amount of Earth’s surface covered by mining waste range from 31,000 to >1,000,000 km^2^ globally.^117−119^ Recent efforts to map mine waste focus mainly on larger-scale areas; less-obvious mine waste, such as those undergoing revegetation, are likely underestimated,^117^ yet these are sites most vulnerable to wildfire. Increasingly accurate mapping of mine sites that includes targeted commodities, mine feature classification, ore body type, and other salient characteristics^120,121^ will aid in assessment of compound event hazards to water quality from wildfire and mining.
- *Expanded monitoring and conceptual understanding*. There are many gaps in postwildfire water-quality monitoring,^29,33,122,123^ particularly for metals and during storm events, that need to be addressed. In addition, very little research has been directed at understanding metal fluxes during individual high-flow events in legacy mined areas.^25^ Other needs include downstream tracking of fate and transport of metals in dissolved and particulate forms; clearer identification of relative contributions of surface versus subsurface metals mobilization processes, and links between these two pathways; and understanding of geochemical processes in deposited sediments in reservoirs and floodplains.
- *Improved modeling*. Risk assessments of compound events of wildfire and mining would benefit from more holistic, process-based approaches. Combining postwildfire water-quality models that include sediment erosion, transport, and deposition from headwaters to critical water supplies^124,125^ with models that incorporate geochemical and biogeochemical processes^111,126−128^ would more accurately represent the combined influences of wildfire and mining.
- *Develop compound event mitigation strategies*. Blended landscape mitigation strategies will benefit treatment approaches for minimizing erosion from the legacy mining waste^21,129^ and burned areas.^130^
